# The impact of COVID-19 on emergency medical service-led out-of-hospital cardiac arrest resuscitation: a qualitative study

**DOI:** 10.29045/14784726.2022.09.7.2.8

**Published:** 2022-09-01

**Authors:** Ali Coppola, Kim Kirby, Sarah Black, Ria Osborne

**Affiliations:** South Western Ambulance Service NHS Foundation Trust ORCID iD: https://orcid.org/0000-0003-0135-3783; South Western Ambulance Service NHS Foundation Trust; University of the West of England; South Western Ambulance Service NHS Foundation Trust; South Western Ambulance Service NHS Foundation Trust

**Keywords:** COVID-19, emergency medical services, out-of-hospital cardiac arrest, resuscitation

## Abstract

**Background:**

Following the emergence of COVID-19, there have been local and national changes in the way emergency medical service (EMS) staff respond to and treat patients in out-of-hospital cardiac arrest (OHCA). The views of EMS staff on the impact of COVID-19 and management of OHCA have not previously been explored. This study aimed to explore the views of staff, with a specific focus on communication during resuscitation, resuscitation procedures and the perception of risk.

**Methods:**

A qualitative phenomenological enquiry was conducted. A purposive sample of n = 20 participants of various clinical grades was selected from NHS EMS providers in the United Kingdom. Data were collected using semi-structured interviews, transcribed verbatim and inductive thematic analysis was applied.

**Results:**

Three main themes emerged which varied according to clinical grade, location and guidelines.

Decision making: Staff generally felt supported to make best-interest termination of resuscitation decisions. Staff made informed decisions to compromise on recommended levels of personal protective equipment (PPE), since it felt impractical in the pre-hospital context, to improve communication or to reduce delays to care.

Service pressures: Availability of operational staff and in-hospital capacity were reduced. Staff felt pressure and disconnect from the continuous updates to clinical guidelines which resulted in organisational change fatigue.

Moral injury: The emotional impacts of prolonged and frequent exposure to failed resuscitation attempts and patient death caused many staff to take time away from work to recover.

**Conclusion:**

This qualitative study is the first known to explore staff views on the impacts of COVID-19 on OHCA resuscitation, which found positive outcomes but also negative impacts important to inform EMS systems. Staff felt that COVID-19 created delays to the delivery of resuscitation, which were multi-faceted. Staff developed new ways of working to overcome the barriers of impractical PPE. There was little impact on resuscitation procedures. Moving forwards, EMS should consider how to limit organisational change and better support the ongoing emotional impacts on staff.

## Background

In March 2020, the World Health Organization declared a global health emergency pandemic due to the rise in the death toll caused by SARS-CoV2 (COVID-19) (World Health Organization, 2020). COVID-19 was a new infectious disease which typically causes mild to moderate respiratory illness. However, patients with underlying health conditions were at risk of developing a serious illness, estimated at 25% of the United Kingdom’s population (Jordan et al., 2020). As of November 2021, in the United Kingdom over nine million people tested positive and nearly 167,000 died having COVID-19 reported as the cause of death (GOV.UK, 2020). Following the emergence of COVID-19, emergency medical services (EMS) in the UK were provided with guidance for practice by Public Health England (2020).

This guidance outlined specific precautions to be taken by EMS staff when undertaking resuscitation as a treatment for patients in out-of-hospital cardiac arrest (OHCA). In the United Kingdom in 2020, 31,368 people suffered an OHCA which was treated using resuscitation delivered by EMS (OHCAO Project Team, 2020). Procedures for donning and doffing personal protective equipment (PPE) and the required levels of protection were outlined.

OHCA presents a number of challenges given the time-critical nature of resuscitation (Linderoth et al., 2015). To optimise successful resuscitation early and effective chest compressions, defibrillation, drug therapies and complex clinical and ethical decision-making are required. Prognosticating patient outcome and safety also needs to be considered when transporting patients in OHCA with ongoing resuscitation (Ong et al., 2018). Resuscitation in patients with OHCA has been complicated by the emergence of COVID-19. As new information emerged, national and local EMS guidelines responded with updated information and revised clinical practice. This study aimed to explore the views of EMS staff on the impacts of COVID-19 on resuscitation, to include communication, resuscitation procedures and the perception of risk.

## Methods

An interpretative phenomenological approach was used to explore the experiences of EMS staff on the impact of the COVID-19 pandemic on OHCA resuscitation. Phenomenology is a unique and powerful strategy that allows learning through the exploration of individual lived experience (Neubauer et al., 2019). This method was selected to reflect theme development derived from participant experience and reflection of that experience to enable an in-depth understanding of the topic area. The study protocol was designed according to the consolidated criteria for reporting qualitative research (Booth et al., 2014).

Staff from UK EMS providers who had performed resuscitation during the COVID-19 pandemic were invited to participate. Study information was sent to contacts from the National Ambulance Research Steering Group for each UK EMS to advertise using internal service methods to ensure participant interest was captured in non-social media users. Social media platforms Facebook and Twitter were also used.

A sample of n = 20 was considered suitable to provide rich data for theme development (Clarke & Braun, 2014). Purposive sampling was employed as paramedics, nurses, technicians, emergency care assistants, students and community first responders have the features required to enable a detailed exploration of resuscitation experiences (Ritchie et al., 2013). Given the wide-ranging skills employed in OHCA resuscitation, maximum variation sampling ensured themes were identified across a variety of clinical staff from various locations. Operational EMS staff were eligible for inclusion if they had performed advanced life support to meet the objectives of this study. Participants were provided with an information sheet and written consent was required prior to interview.

Semi-structured interview questions developed by the research team were used to collect data (Supplementary 1). This method was chosen to enable participants the freedom to elaborate on answers (Clarke & Braun, 2014). Focus groups were not considered appropriate due to the underpinning phenomenological approach. As social distancing measures and travel restrictions were in place, interviews were completed virtually and were audio-recorded by the lead author. Field notes were taken by an operational paramedic with an interest in research.

Data were analysed using inductive thematic analysis to focus on theme development as data were not shaped by existing theory. The six phases of thematic analysis were conducted (Clarke & Braun, 2014): (1) familiarisation, (2) generating codes, (3) generating themes, (4) reviewing themes, (5) defining and renaming themes and (6) the report. Interview transcripts were allocated non-identifiable codes, anonymised, transcribed verbatim and organised using NVivo (version 12) software.

Data were cross-coded by two reviewers (AC, KK) and disagreements were resolved by consensus. The relationship between the data generated, the participant and the author was reflected upon. The lead authors of this study are operational paramedics who worked throughout the pandemic. Personal reflexivity was undertaken to enable the authors to consider the potential for bias when interpretating the results for theme development. A journal was used to increase self-awareness and reduce personal influence when writing up the study findings.

## Results

Twenty staff from eight UK EMS were interviewed between April and June 2021. Interviews lasted approximately 45 minutes. Participant demographics are shown in Table 1.

**Table 1.** Demographics of participants.

**Age**	Median: 33 years IQR: 14.5 years
**Role**	Technician: 3 Student paramedic: 1 Paramedic: 5 Newly qualified paramedic: 2 Clinical team manager, paramedic: 1 Clinical supervisor, paramedic: 2 Advanced paramedic: 1 Critical care paramedic: 1 Consultant paramedic: 1 Community first responder: 1 Emergency care assistant: 2
**Length of service**	Median: 7 years IQR: 10.5

IQR: interquartile range

Note: Gender was recorded, yet excluded as a participant demographic to maintain the confidentiality of those who took part.

Table 1 demonstrates a range of participant ages between 21 and 64 years. Length of service between 1.5 and 43 years and 11 different roles were represented. Titles of roles varied between each EMS, specifically for registered paramedics which ranged from newly qualified to consultant level.

### Themes

**Figure 1:**
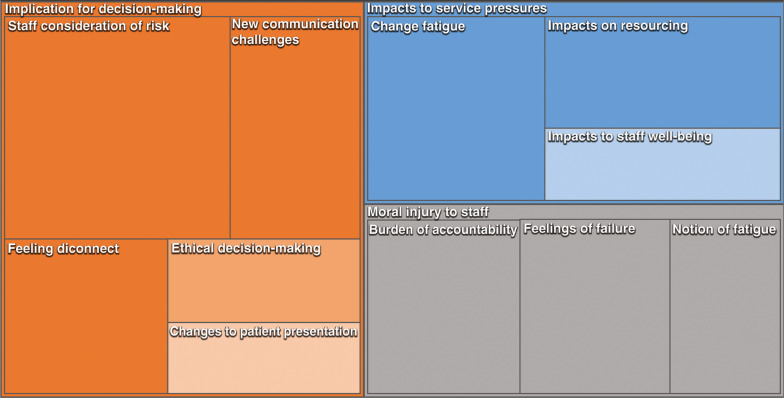
Figure 1. Themes and sub-themes treemap of coding references.

Three main themes are illustrated in the treemap of coding references (Figure 1). These are: theme one, implications for decision making; theme two, impacts to service pressures; and theme three, moral injury to staff. Coding reference values are shown in Supplementary 2.

#### Theme one: implications for decision making

##### Staff consideration of risk

In practice, the lines between personal risk and delivering timely treatment became blurred when managing resuscitation, particularly when the event was witnessed.

If a patient goes into cardiac arrest on you, that’s the hard bit. In all honesty, I stuck an i-gel down with only level two PPE on. Which I know goes against the guidelines, but what else do you do? Oh hang on a minute, I know you’ve gone into cardiac arrest, let me just put a Tyvek suit and powered hood on. (T11CPAS)

Patient care over personal risk was particularly felt in paediatric and young people.

Cases that I can think of are paediatric choking, where I didn’t wear any PPE. There’s been a conscious decision to not spend that time donning PPE. (T13 SAAS)

Staff attempted to reduce the risk of transmission as much as possible. There was awareness that adequate PPE reduced transmission; however, many participants felt the provision of PPE was not practical for OHCA. Participants felt aprons were more likely to increase the risk of transmission and were unsuitable when managing OHCA resuscitation.

The aprons that we’re given as well are just really inappropriate for pre-hospital care. They’re really thin and they blow up into your face if you go outside. So actually, if anything, it’s putting COVID-19 on you. (T12TCAS)

##### New communication challenges

Communication was challenging, mostly due to the barriers of PPE. Using radios was difficult. Staff described shouting to be heard, exposing families to sensitive conversations.

We need to make a decision whether to call it. And that’s when the family then screamed their heads off and went into the kitchen, cos they could hear me. But I couldn’t say it any other way. (T7FPAS)

New ways to communicate were found. Staff became focused and efficient, making themselves heard and using badges to identify names and clinical grades.

It’s probably put the right focus on communication. If I’m honest it’s probably the method of communication we should be doing. We probably always haven’t done, but by necessity we’ve been forced into really focusing on that, really making it part of our plan. (T17NCAS)

##### Feeling of disconnect

EMS staff described a feeling of disconnect, resigned to letting patients and relatives down, mostly due to a sense of duty conflicting with organisational policy. A disconnect was also felt between clinical decisions and in-hospital staff. There was a perception that the number of patients who had resuscitation efforts ceased on arrival had increased.

Patients seem to be called earlier in hospital and I’ve had one called as soon as they’ve arrived at hospital, without even getting off the vehicle, cos they had no space. So yeah, that’s been different. (T12TCAS)

##### Ethical decision-making

Staff felt more decisive to make ‘best interest’ resuscitation decisions. These decisions included early termination of resuscitation or recognition of life extinct for patients where continued resuscitation was felt to be of no benefit.

It feels like we’ve made decisions more solidly than perhaps we would’ve done previously. Obviously if there’s a TEP or DNR in place, it’s fine. But perhaps, situations where there’s history and we might’ve ummed and erred, we’ve possibly been more decisive about it. (T8SEAS)

Generally, staff felt well supported by their employing organisation, with a greater level of autonomy resulting in confident decision-making compared to pre-pandemic times.

Under our normal guidelines, a shockable rhythm we’d have loaded and gone to hospital, to get there for the doctor to call it. Having the autonomy to actually make those decisions and having the confidence to make those decisions for out-of-hospital cardiac arrest, I actually prefer it. (T11CPAS)

##### Changes to patient presentation

Staff perceived an increase in non-shockable rhythms, and in suicide and cardiac arrest in younger patients.

There was an obvious shift from going to workable cardiac arrests to going to a lot more people who were dead on arrival, and younger patients, during the first wave. During the second wave noticeably more PEA. (T12TCAS)

Staff also felt that they were sent to patients with little hope of survival when preventative deaths were left waiting.

I felt that we were wasting a lot of time doing that, that we could’ve been out helping these patients that weren’t getting ambulances and subsequently dying. (T20TCAS)

#### Theme two: impacts to service pressures

##### Change fatigue

Change fatigue described the rapid and continuous change in the clinical management of patients, PPE and organisational policy. Staff understood amendments to policy due to the rapidly changing nature of COVID-19, but many changes were felt to be unnecessary.

We’ve absolutely been deluged with notifications, updates; this, that and the other government guideline is changing. National ambulance guidelines are changing, local trust guidelines changed. It’s been a bit torturous. (T3CTAS)

In practice, new policies had little impact. Staff felt treatment pathways remained the same and there was a disconnect between actual clinical practice and the expectation of policy.

Constant updates and policy changes; that was a lot to get with. There were times that I wouldn’t even bother looking at them because I’d think I’m just absolutely fed up with all these new policies coming out. I’d just take the patient into hospital; I’d just do what I’d been doing before. (T7FPAS)

##### Impacts on resourcing

Staff felt organisational resources were under pressure due to increased service demand and the availability of staff due to contracting the virus, isolation rules or burnout. Staff felt patient outcomes declined as reduced operational resourcing led to delays to care.

I’ve seen significantly more cardiac arrests than I would do usually. And I’m also seeing a lot of patients, when it’s been busy, patients that should’ve had an ambulance response an hour or two prior to them having a cardiac arrest. Which is quite difficult from a family point of view, because we should’ve been there earlier and now they’ve died. (T12TCAS)

##### Impacts to staff well-being

Staff felt that COVID-19 impacted on their well-being at work, which for some led to impacts at home. One participant described the increased and prolonged exposure of critically unwell patients as the main cause for a decline in their well-being. Many staff reported taking time away from work to recover.

I think, psychologically, for a lot of people, specifically myself, I had a pretty bad run of pretty sick people. And pretty big cardiac arrests. I think for me, I definitely noticed a difference in my practice, but I’ve also noticed a difference psychologically. I’ve had to shout for help since. (T6MTAS)

Staff well-being was also impacted when conflict was experienced with family members, particularly when religious ceremonies following patient death were not held due to social distancing measures which caused distress to all.

There was no availability for the family members to be able to carry out their religious formalities, we faced quite a bit of backlash. Which is kind of a bit of a kick in the guts, that we did this to people for no reason. It was a difficult time. (T9SPAS)

#### Theme three: moral injury to staff

##### Burden of accountability

Staff were very aware of the emotive impacts of the increased exposure to resuscitation and death. Overall, staff felt better supported to cease resuscitation than they did prior to COVID-19. These decisions came with a burden of accountability impacted by PPE fatigue, the perception of more patients deteriorating into cardiac arrest with failed resuscitation attempts, breaking bad news and managing family expectations.

The increased number of deaths, the increased number of rules, and the increase in contact with public who deny the existence of COVID or, you know, abuse us or whatever. It has been hard. (T3CTAS)

##### Feelings of failure

Participants described a feeling of failure to provide the best care and achieve a successful patient outcome. Staff perceived that more resuscitations were terminated at the scene and this increased their exposure to the deteriorating patient and death.

I think we all felt like a bit of a failure as paramedic or a failure as an ambulance service clinician at the time, because we weren’t providing the care that we usually would or that we were able to. (T9SPAS)

##### Notion of fatigue

Resuscitation fatigue was felt by staff, even when resuscitating patients with factors known to optimise survival.

ALS hasn’t changed during it. I think it’s just more the staff attitude rather than the care the patients get. Maybe we are not starting resuscitation on more than we would before. Maybe that’s more in our mind that is there actually any point in starting resuscitation and putting them and the family through a brutal process, really. For not a positive outcome. (T14NPAS)

Staff also described the effects of compassion fatigue when performing resuscitation in PPE. One participant described a resuscitation attempt which raised serious concerns about crew safety.

I remember that day where I did four arrests, it was like 34-degree heat. It was just a nightmare, poor old crews were just sweating buckets. We had one member of staff, we’d finished this arrest and she knelt on the ground, and I said, are you OK? She said I’m going to pass out. (T10MSAS)

## Discussion

This is the first known qualitative study to explore the views of EMS staff on the impacts of COVID-19 on resuscitation. These findings are similar to those of previous quantitative impact studies: delays in response times due to service pressures (Yu et al., 2021), donning PPE, disruption to healthcare services (Scquizzato et al., 2020) and increased exposure to death (Lim et al., 2021). The findings of this study look beyond these impacts which help us to understand change fatigue, the barriers of PPE, new ways of working for effective communication, confidence and ‘best-interest’ decisions and the personal risk taken by staff for patient benefit.

Staff reflected on the persistent updates to national and local guidelines. These resulted in change fatigue; staff saw little benefit to the updates and stopped reading them, feeling a disconnect with policy makers. This finding is congruent with McMillan and Perron (2020), who found that in healthcare, change fatigue created a culture of exhaustion, poor practice and a demotivated workforce.

EMS providers applied PPE guidelines from Public Health England. Despite this requirement and acknowledged barrier to infection, staff felt that PPE was not practical for OHCA resuscitation. Aprons were described as flimsy and were thought to increase rather than decrease the transmission of infection. Staff highlighted PPE as a challenge to effective resuscitation, causing fatigue, particularly when performing chest compressions. This view aligned well with previous research which found that PPE significantly decreased effectiveness (Chen et al., 2016). Staff found PPE a barrier to communication and described communicating with relatives as challenging, particularly when discussing best-interest decisions or breaking bad news. Participants felt these challenges further increased the emotional stress to relatives. No pre-hospital studies on this topic area were found. In hospital, breaking bad news during COVID-19 was explored, finding minimal PPE and regular sanitation adequate to support relatives (Tikka et al., 2020). This approach is not transferable to the practicalities of the pre-hospital context (Vikke et al., 2019).

To manage these barriers, new ways of communication emerged. Participants used stickers to identify their names and clinical grade, reducing the risk of delegating tasks beyond the scope of clinical practice. Staff described that clear and succinct communication reduced noise and improved shared decision making. This finding supports Cormack et al. (2020), who found that good communication positively impacted team performance, which reduced the risk of error.

Staff experienced an increased level of autonomy to make resuscitation decisions and felt well supported, with advanced paramedics available for advice. A study by Anderson et al. (2018) found that paramedics required confidence and experience to make resuscitation decisions. In this study, paramedics experienced an increased exposure to resuscitation. This exposure increased the confidence of staff, who described a lower threshold for terminating resuscitation compared to pre-pandemic times. There was a perception of disconnect between EMS staff and in-hospital practice. Staff felt that patients conveyed to the hospital with ongoing resuscitation experienced treatment being withdrawn when typically treatment would have continued. This impacted on staff and they described feelings of failure which contributed to moral injury.

Staff reflected upon the prolonged and increased exposure to death, feelings which describe compassion fatigue and an increased burden of accountability. EMS was previously identified as a workforce at high risk of moral injury, mostly due to the disconnect between personal values and organisational expectation (Lentz et al., 2021). In this study, we further identify the burden felt by participants, their feelings of failure and inability to achieve a personal and professional standard of care. Moral injury in paramedicine is not a new concept; it does however require adequate support from EMS providers (Murray, 2019). Participants highlighted that many staff took time off work to recover their well-being. This finding reflects Greene et al. (2021), who predicted that nearly 58% of healthcare workers would experience post-traumatic stress disorder and anxiety due to the pandemic.

Personal risk for patient benefit impacted greatly on staff. EMS staff were considered seven times more at risk of developing severe COVID-19 illness due to close patient contact (Mutambudzi et al., 2021). Staff appeared to carefully consider and weigh the risks; however, in a witnessed collapse or with paediatric or young patients, staff felt conflicted as time delays to care are known to reduce survival (Banerjee et al., 2021). Staff reduced delays to starting resuscitation by remaining in level two PPE as they felt the benefit in these patient groups outweighed their personal risk.

### Study strengths and limitations

The sample size applied in this study was guided by the methods required to generate new knowledge. Participants had a ‘rich’ experience of the phenomenon and generated in-depth data for theme development to meet the study aims. This approach is supported by Malterud et al.’s (2016) concept of ‘information power’. The purposive sample provided a breadth of personal experience to be explored. While participants demonstrated variation in characteristics, the study focus was narrow. The interview dialogue held focus on the pre-defined questions; however, the semi-structured nature encouraged the participants to answer openly, elaborating to further explore important points of discussion. The interview discussions were clearly communicated, with good interaction between the author and participants. However, in the national and international context, findings may not be generalisable across all EMS systems as this study represented the views of staff from eight of the 14 UK providers. EMS providers typically operate locally amended resuscitation guidelines which may have impacted the resuscitation decisions made by staff. The potential for bias was previously acknowledged; however, the findings of this study demonstrate internal validity reaching sufficient ‘information power’ to offer new insights to improve our understanding of the impacts of COVID-19.

## Conclusion

EMS views on the impact of COVID-19 on OHCA resuscitation varied. There are positive outcomes but also negative impacts, and these findings are important to inform EMS systems. COVID-19 created delays to OHCA resuscitation which were multi-faceted. EMS staff developed new ways of working to overcome the barriers of PPE on communication during resuscitation. There was little impact on resuscitation procedures. EMS staff made informed decisions in certain patient groups to reduce delays to resuscitation caused by donning PPE or limited resource availability. Organisational change fatigue contributed to the moral injury of staff. EMS providers should consider how to limit organisational change and better support the emotional responses of staff with increased and prolonged exposure to OHCA resuscitation.

## Acknowledgements

The authors would like to thank the contributors and participants for their valued input throughout this study, as well as Megan Haskin for assisting with field notes and Gail Thornton for transcription.

## Author contributions

Study concept and protocol was written by AC and KK. Interviews were undertaken by AC. Coding and theme development was conducted by AC and KK. Results write-up was led by AC and revised and edited by KK and SB. AC, KK, SB and RO reviewed and edited the study results for publication. SB acts as the guarantor for this article.

## Conflict of interest

None declared.

## Ethics

Approval was obtained from the UK Health Research Authority (IRAS Project ID 286001). The study was adopted by the NIHR portfolio (CPMS ID 47990).

## Funding

This study was funded by the college of paramedics grant scheme (£1250) and by research capability funding from the South Western Ambulance Service NHS Foundation Trust (£1250).
